# Establishing Trauma-Informed Primary Care: Qualitative Guidance from Patients and Staff in an Urban Healthcare Clinic

**DOI:** 10.3390/children9050616

**Published:** 2022-04-26

**Authors:** Andrea Matthew, Cynthia Moffitt, Alissa Huth-Bocks, Sarah Ronis, Mary Gabriel, Kimberly Burkhart

**Affiliations:** 1 Department of Pediatrics, UH Rainbow Babies & Children’s Hospital, Cleveland, OH 44106, USA; andrea.matthew@uhhospitals.org (A.M.); alissa.huth-bocks@uhhospitals.org (A.H.-B.); sarah.ronis@uhhospitals.org (S.R.); 2 Division of Pediatric Critical Care, Nationwide Children’s Hospital, Columbus, OH 43205, USA; cynthia.ar.moffitt@gmail.com; 3 School of Medicine, Case Western Reserve University, Cleveland, OH 44106, USA; mary.gabriel@uhhospitals.org; 4 Center for Child Health & Policy, UH Rainbow Babies & Children’s Hospital, 11100 Euclid Ave MS 6036, Cleveland, OH 44106, USA; 5 Department of Psychiatry, University Hospitals Cleveland Medical Center, Cleveland, OH 44106, USA

**Keywords:** trauma-informed care, qualitative, patients, providers, primary care

## Abstract

Patients present to primary care clinics with a variety of experiences, including exposure to adverse childhood experiences (ACEs) and other social determinants of health. The pervasive impact of early adversity on later healthcare outcomes has resulted in the development of trauma-informed care principles that can be applied to healthcare settings. The primary aim of this study is to improve understanding of patient and staff experiences within a trauma-informed urban healthcare setting to guide considerations and recommendations when implementing such a model. A phenomenologic approach was taken using an interpretivist paradigm to collect qualitative data by conducting patient and staff focus groups. The following themes were identified: the communal experience of significant trauma, lack of continuity of care and time for each appointment, the importance of a sense of community and standardization and normalization of asking about trauma, development of social support networks, and creating a safe and non-judgmental healthcare space. Based on findings, considerations for implementing a trauma-informed healthcare model are provided.

## 1. Introduction

Patients present to primary care appointments with a variety of experiences, which could serve as risk factors for poor health outcomes. A biopsychosocial assessment approach is recommended to increase understanding of how current health presentation may be impacted by ACEs (e.g., child maltreatment, abuse, parent divorce/separation, substance use by parent, and/or an incarcerated parent) or other social determinants of health [1,2], hereafter broadly referred to as ‘childhood adversity’ [3]. Large-scale studies have established that childhood adversity is highly prevalent, with estimates suggesting that 60% of US adults [4] and up to 45% of US youth [5] have experienced at least one ACE. The impact of childhood adversity on physical and mental health problems has also been shown to persist into adulthood [6]. Research has demonstrated, for example, that exposure to childhood adversity is associated with increased risk of many chronic diseases with high morbidity and mortality, including cancer, coronary artery disease, asthma, kidney disease, and cancer [4,7]. Childhood adversity is also associated with increased involvement in mental health and substance use services, increased risky behaviors, such as tobacco and alcohol use, increased learning and behavioral problems, as well as negative impacts on education and employment [4,7,8]. In one meta-analysis, the population-attributable risk associated with childhood adversity was highest for anxiety and depression, confirming the existing concern that individuals who experience trauma are at much higher risk for negative mental health outcomes [9].

The accumulated evidence about the pervasive impact of early adversity on later health outcomes has resulted in advocating for a trauma-informed care model to be implemented into medical practice, as evidenced by guidelines developed by the Substance Abuse and Mental Health Services Administration as well as the National Council for Behavioral Health [10,11]. “Trauma-informed practice” and “trauma-informed care” both refer to a program, organization, or system that is intentionally designed to support individuals or groups of individuals who experience adversity that could be experienced as traumatic [12,13]. Implementation of trauma-informed care may be particularly important in healthcare settings that serve low-income patients and/or patients self-identifying as a race (including multi-racial) that have been systematically marginalized, as research has repeatedly documented that serious childhood adversity is disproportionately over-represented in these groups [14,15,16].

The trauma-informed approach reinforces the importance of understanding what a patient has experienced and how this might affect their physical and psychological well-being, ultimately with the goal of providing effective and sustainable care [11]. Trauma-informed practice in medicine is particularly relevant given that patients who have a history of adversity are often re-traumatized and experience distress during interactions with healthcare providers and systems [17]. Patients may be distressed by inappropriate questions asked of them, insensitive responses to their disclosures, or may simply lack the rapport with providers needed to feel safe engaging in such conversations. Trauma-informed care attempts to mitigate re-traumatizing or problematic patient-provider interactions by grounding practice within a broader safe, supportive environment that acknowledges the pervasive nature and effects of childhood adversity, and furthermore, where providers are trained to compassionately and sensitively address adversity in an intentional and culturally-competent way [10,11].

Screening for childhood adversity in both adult and pediatric care settings is one important part of a trauma-informed care model; however, it is well recognized that screening must be done within the context of a broader trauma-informed setting [3,18]. Other important components of an effective trauma-informed care setting include: an assessment of clinic readiness and knowledge, provider/staff training, development of a screening response protocol, expansion of brief trauma-informed interventions and/or linkage to trauma-informed services, and a focus on ongoing provider/staff wellness.

Prior studies on acceptability and feasibility of childhood adversity screening in primary care has indicated that, overwhelmingly, patients find this type of screening appropriate and acceptable in the context of healthcare visits. For example, several studies found that most adult patients (>90%) were comfortable answering these sensitive questions about themselves, and parents strongly supported screening by their children’s providers, viewing their pediatrician as an important change agent [19,20]. Other studies reported that adult patients felt positive about being asked about their history of adversity and perceived that clinicians could offer help, and that the act of asking improved the patient-provider relationship [21,22]. Moreover, providers indicated that pediatric and family medicine clinic visits were not unduly burdened by adversity screening in terms of time or patient resistance, and visit length increased by only approximately 5 min or less 90% of the time [23,24].

Despite empirical support for the acceptability and feasibility of childhood adversity screening within primary care, there has also been some debate and concern regarding “universal screening” and about the role and feasibility of screening, particularly in settings with complex multi-need patients and limited visit time. More specifically, one such concern is that the original Felitti and Anda 10-item ACEs questionnaire excludes important adversities, such as social factors like poverty and racism [25]. Moreover, adversity screening tools, such as the ACEs questionnaire, do not identify protective factors or factors that might promote resilience [25]. There are also many concerns regarding costs and the potential negative effects of screening. Costs may be related to time and effort spent on screening, training required to properly screen, and overtreatment in response to positive screens that might not have been necessary [26]. Additionally, there is concern about possible distress induced in patients from screening and possible disruption of the patient-provider relationship from screening [26]. Thus, it is important to further understand the knowledge and perspectives regarding addressing screening and responding to patient childhood adversity from both patients and staff using a qualitative-centered lens.

The primary aim of this study was to solicit perspectives and opinions grounded in patients’ and providers’/staff lived experience within a trauma-informed care model in an ambulatory healthcare setting serving women and children. Of particular interest, we sought to obtain additional information on perceived benefits, challenges/barriers, and processes that are viewed as helpful when implementing a trauma-informed practice including screening for childhood adversity. Gathering these perspectives was part of our center’s efforts at assessing setting knowledge and readiness for trauma-informed care, one important aspect of the model as noted above. Information gained helps inform screening and other aspects of programmatic planning, including the development of a response protocol to screening results. Further, by reporting on responses from both adults who access services for themselves and their children as well as health care staff, this qualitative study seeks to serve as a resource for healthcare settings, especially other ambulatory practices serving women and children, by providing considerations and suggestions for implementing a trauma-informed practice.

## 2. Materials and Methods

### 2.1. Study Design

An interpretive phenomenologic qualitative approach was taken for the purposes of exploring the complex topic of trauma and trauma-informed care within healthcare settings [27]. The study was approved by the Hospital’s Institutional Review Board (IRB), and all regulations were followed for the ethical treatment of human subjects. Focus groups were conducted with patients, providers, and staff at a large urban ambulatory care practice serving women (ob/gyn) and children (pediatric primary care). This practice serves as a training site for 80–100 pediatric and ob/gyn residents combined. Acknowledging the difficulties of extrapolating individual voice from group settings [28], we selected this approach to ensure that the process of reflexivity would manage research team members’ preconceptions on the topic [29], while integrating the influences of local culture and context into our understanding of how various trauma-informed approaches might be received in this setting [30]. In opting for focus groups rather than individual interviews, we sought to balance our goal of eliciting the meanings individuals ascribe to experiences of trauma with the opportunity to illuminate group dynamics that could be expected to emerge within the multidisciplinary clinical team being asked to implement a trauma-informed model at our site. Furthermore, focus groups are helpful in exploring sensitive topics where conversation in a group depersonalizes the experience of storytelling.

The study planning team consisted of experts in maternal and infant mental health, trauma-informed psychotherapy, psychiatry, and primary care physicians working within the trauma-informed care model at the clinic. Focus groups were facilitated by a trained medical anthropologist and an expert in maternal-infant mental health, neither of whom provide direct care to patients nor have supervisory responsibilities for the participating employees. Field notes were taken at the time the focus groups were conducted.

### 2.2. Participants

The academic urban primary care clinic where this study took place houses innovative programs for pediatric primary care, women’s health care, and wrap-around social services as well as educational programs to support patients, their families, and the community. The clinic serves under-resourced individuals from some of the poorest urban neighborhoods surrounding the clinic. Recent analysis of center patients revealed that 91% of patients identify as African-American/Black with a median household income of $29,000. Approximately 95% have public health insurance (Medicaid). Intentional efforts are made to employ individuals that also represent the community; recent analysis of clinic employees showed that about 66% identify as African-American/Black, and 91% are female. Characteristics of participants in the present study (*N* = 30 employees, *N* = 6 mothers receiving health services for themselves in the women’s health clinic and/or pediatric services for their children) are shown in Table 1; no other demographic characteristics were collected from employees or patients in an effort to increase comfort with sharing perspectives in the groups. However, participants are believed to represent clinic patients and clinic staff.

### 2.3. Procedures

Focus groups were conducted with providers/staff and patients as part of a comprehensive trauma-informed care implementation, including the preparation for a practice wide childhood adversity screening initiative using the ACEs-Q [8], Protective and Compensatory Experiences (PACES) screen [31], and the Safe Environment for Every Kid (SEEK) screen [32,33] at well-child visits through age 6 years (parent/caregiver report) and an expanded Philadelphia-ACEs screen for patients in women’s health [34]. At the time of this study, providers and staff also began receiving training on trauma-informed care principles and practices at regularly scheduled staff meetings.

Health care staff were recruited through a center-wide email distribution list. The email script described the purpose of the focus groups and read, “The purpose of the focus group is to better understand people’s experiences working at the (name of ambulatory healthcare clinic) in regard to patient adversity and trauma”. Six different focus group sessions were offered with different days and times, including early morning, mid-day, and evening. Individuals interested in participating contacted the lead investigator of the study and attended the group that fit best with their schedule.

Seventy-five patients receiving care at the health care center, who had previously given permission to be contacted for research, were called on the phone (each up to two times) by study staff. Like employee groups, patients were given a choice of days/times to attend a focus group; four offerings were provided to patients. The purpose of the group for patients was stated as “The purpose of the focus group is to better understand patients’ experiences with clinic staff and of receiving care from clinic providers. We are interested in your thoughts about how clinicians should provide care to those who have experienced adversity, stress, or trauma”.

Participants in all groups were sent a copy of the informed consent document ahead of time through email and then were given the document in person at the time of each group, after which they had a chance to review it and ask questions before signing. Written consent was provided for audio-recording as well. All groups lasted approximately 60–80 min. Light food and refreshments were served at all focus groups, and patients each received a $10.00 gift card as compensation for their time. See Supplementary Text S1 and S2 for employee and patient focus group questions, respectively.

### 2.4. Data Analysis

An immersion/crystallization approach to content analysis was taken [35]. First, all focus group recordings were transcribed verbatim by a professional transcribing service. Recordings and transcripts were stored on the hospital-encrypted server and shared only with members of the research team. Second, three members of the research team (one clinical psychologist and two pediatric physicians) immersed themselves in the data through detailed review of the complete focus group transcripts and their accompanying field notes. Next, reviewers independently developed codes to organize the data, grouping related codes to capture emerging themes and subthemes. At that stage, the reviewer team came together to reflect on the influence of their own perceptions and biases in their coding the data, bracketing their own pre-understanding of trauma-informed care from the experience as described by focus group participants. Through discussion, the team reconciled differences in their application and interpretation of themes. Once consensus was achieved through group discussions among all three coders regarding themes, subthemes, and their underlying codes, team members together developed a report of the common themes that were shared across focus groups with exemplar quotes to support those themes, as presented in the Results section below.

## 3. Results

After careful review and analysis of transcripts, several recurring themes were identified in the transcripts of both staff and patients within topics elicited by focus group questions. Results are organized by broad topics with identified themes in order of most commonly mentioned to least commonly mentioned. Each identified theme is exemplified by direct quotes from individual participants. Note that while the selected quotes reflect only the opinions of those individuals, the broader themes were identified by reviewers when multiple individuals mentioned them within a group and when themes were apparent across groups.

### 3.1. Topic: Meaning and Impact of Trauma

In response to questions about the meaning and impact of trauma, the most common theme was overwhelmingly that patients at the clinic experience significant trauma which often involves violence and loss. Specifically, patients mentioned shootings, threats, domestic violence, and exposure to murder and other forms of death. Staff also mentioned violence exposure, as well as experiencing the loss of expectant women’s infants or the deaths of mothers seen in the women’s clinic. When discussing these traumas, both sets of participants associated these events and experiences with significant grief as well as safety concerns.

Many participants, patients and staff alike, also reported that trauma frequently results in distress and a sense of urgency. They noted that trauma continues to have an impact after it occurs, which can be related to developmental changes and a myriad of psychological effects. See Table 2 for additional illustrative narrative quotes from different individuals and across groups.

### 3.2. Topic: Barriers to Trauma-Informed Care

The most frequent barrier to trauma-informed care identified by both patients and employees was lack of continuity of care between patients and providers. Participants noted that lack of continuity makes it difficult to form a trusting relationship within which trauma can be disclosed comfortably. For example, new providers are less likely to know the patient’s history and rely on brief chart review to update themselves on a patient’s needs prior to the visit. Patients expressed frustration by the lack of continuity and a desire to know providers over time. They expressed concern that if providers do not know their history and the context of their concerns or living situation, they will be judged, not be helped, or their children might be taken away by Child Protective Services.

The next most common concern was lack of time at each appointment. Staff expressed feeling that they frequently do not have enough time to spend with patients to adequately address their needs or to discuss trauma, nor did they feel there was enough time to process what they experience as providers when caring for trauma-exposed patients. Patients, too, indicated a desire for time, noting that the best thing providers could do when approaching a patient about their trauma is to allow time for them to “open up,” which is not always logistically feasible. See Table 3 for illustrative narrative quotes from different individuals and across groups.

### 3.3. Topic: Facilitators and Recipients of Trauma-Informed Care

In response to questions regarding what our clinic is doing well, the main strength that both patients and staff identified at the clinic was a sense of community. Multiple patients endorsed that they feel comfortable at the clinic and feel like they will be helped. Staff similarly endorsed that they feel that they do a good job of being open to anyone who needs help and that the clinic has become an integrated part of the community it serves. See Table 4 for illustrative narrative quotes from different individuals and across groups.

### 3.4. Topic: Ways to Increase and Improve Trauma-Informed Care

The most common specific recommendation for addressing trauma among staff and patients was standardization and normalization of asking about trauma. Using standardized questions or short questionnaires were noted by both patients and staff as a means of bringing up trauma experiences for discussion. For example, patients could endorse which adversities they had experienced and providers could then follow up regarding their specific responses. Employees acknowledged that it is important for them to know what a patient has experienced in order to address their needs, and patients indicated that they think this would help with communication and better follow-through.

Patients and staff also endorsed that while the center has many resources, there is not always enough to fulfill everyone’s needs. Patients expressed a desire for more basic necessities, and, likewise, staff felt that having more things to offer, such as information on community resources, would help them support their patients. See Table 5 for illustrative narrative quotes from different individuals and across groups.

### 3.5. Topic: Coping Mechanisms

Both patients and staff identified the importance of social support networks, including family and friends, as their main source of coping with stress and motivation to persist despite facing adversities. Both sets of participants indicated that focusing on relationships with children, spouses, and friends or co-workers helps support them and keeps them going. They also identified that these close relationships are what they turn to when they are facing trauma or adversity of their own. See Table 6 for illustrative narrative quotes from different individuals and across groups.

### 3.6. Group-Specific Themes

As can be seen in Supplemental Text S1 and S2, some interview questions were similar across groups; however, some unique questions were presented to employees/staff and patients, respectively. As a result, some unique themes emerged that were specific to each set of participants.

A common theme for employees, specifically, was the significant impact of patients’ trauma experiences on them. They identified that their work could be triggering, exhausting, and frustrating. They also noted being affected by the inability to help in some circumstances. Interestingly, despite acknowledging that they are not always able to help, they described knowing they are helping patients contributes to the ability to cope with the stress and strain related to work. That is, the fulfillment of the work they do, despite the challenges it poses, keeps them going.

A common theme for patients, specifically, was how critical it is for providers to create a safe and non-judgmental space for them in order to be comfortable disclosing trauma. They described wanting to feel like their providers are patient and willing to listen as they discuss such personal issues. They stated that they are much more willing to open up if a provider has an open and compassionate attitude and avoids nagging, “aggressive” questioning and repeated questioning when a patient appears hesitant to share. See Table 7 for illustrative narrative quotes from different individuals and across groups.

## 4. Discussion

The aim of this qualitative study was to solicit perspectives of patients accessing a primary care practice serving women and children, as well as staff working at the practice as part of a larger, comprehensive trauma-informed care implementation. Obtaining qualitative information provides unique insight into individuals’ thoughts and attitudes about sensitive topics, such as life stress and childhood adversity. Our goal in soliciting these lived experiences (as patients receiving care for themselves and their children and as providers of care) was to help inform our center’s childhood adversity screening protocol and other programmatic planning. Results also serve as a vital source of information for the broader field of trauma-informed health care, and especially within pediatric and family healthcare settings, where adversity can be addressed earlier in life, to help optimize patient and staff experience.

After completion of focus groups with both patients (i.e., mothers) and staff about trauma and trauma-informed care at our academic urban health center, qualitative analyses revealed a number of themes in response to interview questions among both patients and staff. Drawing upon prior literature, a priori expected themes included loss and violence, lack of provider continuity as a barrier to trauma-informed care, and relationships as an important protective factor in coping with distress. Consistent with expectations, we found that both staff and patients readily identified forms of loss or violence as a common experience for children and families at the clinic. This included loss due to violence, witnessing violence, or being a direct victim of violence, which are all consistent with national data on common forms of childhood adversity in urban pediatric populations [8,36]. These findings reinforce the importance of training for providers and staff on how to compassionately approach and respond to such conversations with patients, including children and their caregivers [10,11].

Prior to completion of focus groups, we were aware, based on published literature and first-hand clinical experience, that lack of continuity can be a barrier to trauma-informed practice and that consistent provider-patient relationships better result in trust between patients and providers that facilitate disclosure [17]. The theme of relationships with friends and family as an important personal coping tool that we discovered is well-supported by literature that shows close, supportive relationships are a crucial protective factor when facing adversity, including for parents who are raising young children [37,38]. While not as common, emergent themes after completion of qualitative analyses by independent reviewers also included: identification of a sense of community as a significant strength and contributor to both patient and staff comfort with discussing childhood and family adversity, as well as the acceptance of and belief in the importance of standardized screening to identify adversity within the context of healthcare. The latter, in particular, is consistent with other studies demonstrating the acceptability of childhood adversity screening during adult patients’ visits with health care providers [19]; our study extends this finding to services within (urban) healthcare settings for both mothers and their children receiving pediatric care.

In addition to the inquiry about trauma-informed care topics for adult patients receiving their own care and care for their children, a particular strength of this study was the inclusion of both providers/staff and patients operating within the same healthcare center. By including staff in addition to the patients served at the clinic, we were able to compare and contrast responses to extract themes among the two groups of participants who interact with each other as child-family health services are delivered. While patient perspectives are crucial to informing a trauma-informed practice, including individuals working with those families gave us insight into how family adversity affects the employee experience as well. Although our study asked staff about patients’ experiences of adversity, it is important to recognize that staff also have life histories which may include childhood adversity which intersect with patient experiences; future studies need to examine how providers’ and staff’s own experiences may impact the delivery of trauma-informed care.

There were also several limitations to this study, particularly a small sample size and enrollment rate for the patient focus groups. It is possible that our low participation rate is related to our recruitment methods; however, it is also likely that low participation reflects the barriers our patients typically face, such as temporary phone numbers, residential mobility, time to meet for groups, transportation to get to the clinic, etc. While the patient group was notably smaller compared to the employee group, identified themes were derived from examples that were proportional to sample size and represent both groups. It should also be noted that the results of this study may not be generalizable to other settings or clinics that are not urban or inclusive of individuals identifying predominantly as African American or Black. It is recommended that future studies obtain person-centered data about lived experience with other patient populations including those of various cultural, socioeconomic, and racial and ethnic diversity. Future research might also benefit from obtaining perspectives directly from children and adolescents rather than relying on caregiver reports only, although caregiver reports are essential for representing the experiences of young children who are not able to report on their own trauma.

The results of this study have important implications for healthcare settings striving to advance trauma-informed care. First, it is apparent that patient trust and willingness to disclose may be directly related to a number of logistical barriers of typical primary care practices, such as limited appointment times and a sense of feeling rushed. In academic settings in particular, where trainees may rotate in and out of a clinic, lack of continuity may be a very significant barrier to providing consistent care, a cornerstone of trauma-informed practices. For an ambulatory healthcare clinic to be successful in trauma-informed care, these barriers must be addressed. Advocacy for expanded time in visits that are appropriately reimbursed as well as advocacy for support staff who are not productivity-based will be key in addressing limited appointment time. It could be possible, for example, that additional time be allotted for new patients, well-child visits, and/or visits where childhood adversity screens will be conducted. Continuity should be prioritized and frequently evaluated at any primary care center focusing on providing trauma-informed care, as should ongoing provider/staff support to address secondary stress.

While an adversity screening questionnaire, such as the ACE questionnaire or other screens for social determinants of health, is not meant to replace conversation with a trusted provider, screening may afford a standardized and efficient way of checking in about life stress and significant adversity with all patients, including caregivers of children seen in the context of trauma-informed pediatric care. Universal screening can potentially avoid bias by eliminating provider choice of who to screen and can normalize adversity, thereby opening the door for difficult conversations. Screening may also indicate needs to a provider ahead of time, allowing for increased opportunities for anticipatory guidance about coping with adversity and building up relational supports to mitigate the effects of stress on children and families [39]. It is important to keep in mind that our focus group data revealed that adult patients and caregivers of pediatric patients might be hesitant to disclose adversities, such as violence or other forms of trauma, if they are concerned that they will not receive help or support. Therefore, if a primary care clinic plans to begin screening for childhood adversity, it must have processes in place that can support families based on what they disclose as part of a broader trauma-informed setting.

Additionally, the patient experience at each appointment could be improved through relevant training for all staff and ongoing staff support. This training could include greeting patients/families, how to respond when serious adversities are disclosed, as well as de-escalation skills. Prioritizing this kind of training works best with support from clinical leaders and administration. Given the burden of secondary stress in employees, training should also include how employees can recognize and address their own histories of adversity or life stress. Staff support and resources are a critical component in a trauma-informed practice. This study focuses on employee experiences with family-specific adversity, so future studies could focus on the personal histories of the employees and assess how this affects the delivery of trauma-informed care.

When considering training staff in trauma-informed care, there are many implications on education for trainees, specifically medical residents. Medical residents have reported low awareness of the opportunity to screen for trauma and adversity as well as low confidence in doing so [40]. However, studies that have assessed the feasibility of trauma-informed care training specifically for pediatric residents showed increases in favorable attitudes, increases in perceived competence, and decreases in perceived barriers [41].

In sum, a successful ambulatory healthcare practice serving children and families is one that is prepared to address both medical and social needs as the two overlap considerably. Patients, including children and their caregivers, expect providers to be non-judgmental and responsive if they are going to share some of their most personal, and potentially distressing, experiences. Through training and ongoing support to providers and staff, as well as commitment to broader trauma-informed structure and processes, identification of childhood adversity and appropriate supportive interventions can occur early so that childhood development and life-long health consequences of exposure can be mitigated.

## Figures and Tables

**Table 1:** Demographic Characteristics of Study Participants.

	Employees (*N* = 30)	Patients (*N* = 6)
Gender		
Female	30 (100%)	6 (100%)
Male	0 (0%)	0 (0%)
Employee Service Area		
Women’s Health Clinic	18 (60%)	NA
Pediatric Clinic	10 (33%)	NA
Both Women’s & Pediatrics	2 (7%)	NA
Employee Role		
Nurse, Medical Assistant	17 (57%)	NA
Physician or Nurse Practitioner	4 (13%)	NA
Mental-Behavioral Health Staff	3 (10%)	NA
Administrative/Leadership	2 (7%)	NA
Other	4 (13%)	NA

**Table 2:** Meaning and Impact of Trauma.

Theme	Participant	Quote
Violence and Loss	Patient	*“People need to get out in the community and work together to get the guns off the streets.”* *“Where we’re at right now is bad. There’s been a lot of shooting going on.”* *“Safety is the key thing I worry about.”* *“[Trauma] can be grief, it can be loss, stressors, pain, hurt…”*
	Staff	*“We have had several people whose babies have died this week…and so all of those things are traumatic.”* *“Parents abuse their children… and it’s because of trauma that they experienced too as a child.”*
Distress	Patient	*“When I hear trauma, it means something serious…it means something needs to be done as soon as possible.”* *“I just left one place where my house was robbed. …The environment we live in, a lot of people have a lack of respect for each other. And when I say lack of respect, these are kids. These are our future. These are our lawyers, our doctors, our nurses.”*
	Staff	*“Trauma to me can be any action or event that would cause a negative or fearful perception of what’s going on.”* *“Trauma to me is something that causes discomfort, pain, anxiety in one’s life after something has occurred.”*

**Table 3:** Barriers to Trauma-Informed Care.

Theme	Participant	Quote
Continuity	Patient	*“I have a fit when I got to see somebody different…I have to explain everything off to you over again.”* *“That’s my kid’s doctor. Me and her are really close so I don’t’ mind talking to her if something is going on with my kids because I know she will help…people talk to certain people.”*
	Staff	*“The problem is that when we see a patient for the first time, they have no reason to trust you.”* *“I think if there’s continuity, I think parents are more apt to talk.”*
Time	Patient	*“Allow [patients] to vent and get it out even if you know…allow space to get their self together and collect their thoughts.”* *“If they shut down, give them time to open up.”*
	Staff	*“We need to realize that patients take a lot of time, and they need to change the amount of time that anyone has with them.”* *“You cannot help them in a 15 minute appointment.”* *“A lot of times we don’t’ have time…because we got other people coming…it’s rushed…we don’t really have time to sit down really and talk.”*

**Table 4:** Facilitators and Recipients of Trauma-Informed Care.

Theme	Participant	Quote
Sense of Community	Patient	*“You know they’ll help me…they will actually take the time and listen…in essence, you got this home team around you.”*
	Staff	*“I think we’re doing a good job of having an open-door policy…just being visible in the community.”*

**Table 5:** Ways to Improve Trauma-Informed Care.

Theme	Participant	Quote
Standardization and Normalization	Patient	*“I would say make some universal questions that pertains to trauma and experience.”* *“You know, even just having a questionnaire sometimes with just maybe 4–5 questions.”*
	Staff	*“I think screening and finding out about the events…that would be where I would say to start because if we don’t know these things are happening, we can’t help them.”* *“Standardizing and normalizing what you’re about to ask I think is the best way to kind of establish comfort.”*
Community Resources	Patient	*“Given people other places where they can look to, especially help if they have trouble finding clothes for their kids, utility help, farmers markets.”* *“Just give more out for the community.”*
	Staff	*“We tend to know agencies and organizations that are available but are tapped out…maybe reaching out to the community and gathering some more kinds of support.”*

**Table 6:** Coping Mechanisms.

Theme	Participant	Quote
Family and Friends	Patient	*“I look at my kids. My kids are the reason. That’s what gets me up and keeps me going.”* *“I try my best to do what I can do for my kids, because it’s not just about me, it’s about my children.”*
	Staff	*“We all support each other to be honest, I think we have a really really good work family, we all help.”*

**Table 7:** Group-Specific Themes.

Theme	Participant	Quote
Patient Safe Space	Patient	*“I would say…when they’re resistant, just try to work with them. Don’t try to over-talk them.”* *“Don’t be too pushy. Don’t be intrusive. Because if you start coming off shooting 1,000,001 questions, you lost her because it took a lot for her to come in there.”*
Effects on Employees	Staff	*“It can trigger something that happened in our lives, I’ve seen employees have an issue before, it really takes a toll.”* *“You get frustrated, and you have a short fuse ourselves sometimes with patients that I think we try not to show, but we’re all human.”* *“It’s wearing. It’s hard. You can’t keep up. It’s emotionally and physically draining. Your brain is working, your heart is working, you’re physically working.”* *“I think when I can finish a day having helped somebody… that’s what keeps me going.”* *“You know our patients really need the help. And when they get it they really appreciate the help.”*

## Supplementary Materials

The following are available online at https://www.mdpi.com/article/10.3390/children9050616/s1, Text S1: Employee Focus Group Purpose and Questions; Text S2: Patient Focus Group Purpose and Questions.
