# Supplementary material for: Establishing Trauma-Informed Primary Care: Qualitative Guidance from Patients and Staff in an Urban Healthcare Clinic
Source: Children (Basel). 2022 Apr 26;9(5):616. doi: 10.3390/children9050616 (PMC9139306; doi:10.3390/children9050616)
Supplement: Supplementary file 1 [file children-09-00616-s001.zip › children-1677317-supplementary.pdf]

Supplementary Materials

Texts S1: Employee Focus Group Purpose and Questions

Purpose: The purpose of this focus group is to better understand your experiences working at the (healthcare setting-blinded for review) in regard to stress and trauma. For the purpose of this group, when we refer to trauma we are referring to extremely stressful life events that can lead to intense feelings, a sense of insecurity and/or fears about safety. We are interested in your experiences and feelings about stress and traumatic events that occur here at the center, as well as your experiences in working with families who have experienced trauma or adversity in their lives. This information will be used to design a trauma-informed program for this Center and/or inform an assessment of the program's effectiveness.

Questions and Discussion:

<u>What is Trauma and Adversity?</u>

1. What does trauma and adversity mean to you?
2. What are some of the traumas or family adversities you have witnessed or experienced in our clinic? What was that like?

<u>How does Trauma and Adversity Affect Your Work?</u>

3. Have you had patients who are particularly challenging to work with? What made that patient challenging? What was that like?
4. What do you think needs to change in terms of how the center supports patients or employees who experience trauma? Where should we start?
5. What responses from other Center staff to that experience were not helpful?

<u>What are Strengths/Assets/Positive Adaptation?</u>

6. What responses from other Center staff to that experience were helpful? Where or to whom did you turn, if anyone?
7. What keeps you going (or how do you cope with) the stress of your job?
8. What are we doing well? What are the Center's strengths in handling patients with trauma or adversity?

<u>Last/closing question</u>: from everything we discussed here today if there was one thing you could change straight away about Midtown's response to trauma/adversity, what would it be?

Texts S2: Patient Focus Group Purpose and Questions

Purpose: The purpose of this focus group is to better understand your thoughts about receiving care at the (healthcare setting-blinded for review) in regard to life adversity and trauma. For the purpose of this group, when we refer to trauma we are referring to extremely stressful life events that can lead to intense feelings, a sense of insecurity and/or fears about safety. We are interested in your thoughts about how the Center should provide care to those who have experienced adversity or traumatic events. This information will be used to design trauma-informed programming for this Center.

Questions and Discussion:

<u>What is Trauma and Adversity</u>?

1. What does trauma or adversity mean to you?
2. What are the top 5 social concerns you have about your children (or yourselves)?
3. What are some of the traumas that you think families who seek care at our center may have experienced?

<u>How does Trauma and Adversity Affect your Experience at the clinic</u>?

4. How comfortable do you think patients and family members would be to talk about those experiences with providers at the Center?
    a. Which types of providers (nurse, medical assistant, physician, midwife, social worker, psychologist or other mental health provider) might they talk to about their experiences?
    b. Are there other staff at the Center who patients might talk to about their experiences?
    c. What would help them to open up to a provider?
    d. What could a staff member or provider do to make it easier for patients to talk with him or her about their experiences?
5. What type of response would be helpful to a patient or family member who shares a traumatic experience with a provider at the Center?
    a. What type of response should be avoided?
6. What do you think needs to change in terms of how the Center supports patients or family members who experience trauma?
    a. Where should we start?

<u>What are Strengths/Assets/Positives</u>?

7. What is the Center staff and providers doing well? What should we continue?
8. What helps you keep going/helps you cope with personal stress and adversity?

<u>Last/closing question</u>: from everything we discussed here today if there was one thing you could change straight away about the Center’s response to trauma/adversity/hardships, what would it be?
